# Innate immunity based cancer immunotherapy: B16-F10 murine melanoma model

**DOI:** 10.1186/s12885-016-2982-x

**Published:** 2016-12-07

**Authors:** Veronika Caisová, Andra Vieru, Zuzana Kumžáková, Simona Glaserová, Hana Husníková, Nikol Vácová, Gabriela Krejčová, Lucie Paďouková, Ivana Jochmanová, Katherine I. Wolf, Jindřich Chmelař, Jan Kopecký, Jan Ženka

**Affiliations:** 1 Department of Medical Biology, Faculty of Science, University of South Bohemia, České Budějovice, Czech Republic; 2 1st Department of Internal Medicine, Medical Faculty of P. J. Šafárik University in Košice, Košice, Slovakia; 3 University of Michigan Medical Center, Ann Arbor, MI USA; 4 Institute of Parasitology, Biology Centre of the Czech Academy of Sciences, v.v.i., České Budějovice, Czech Republic

**Keywords:** Cancer immunotherapy, Innate immunity, Melanoma, Neutrophils, Resiquimod, Mannan, Phagocytosis

## Abstract

**Background:**

Using killed microorganisms or their parts to stimulate immunity for cancer treatment dates back to the end of 19^th^ century. Since then, it undergone considerable development. Our novel approach binds ligands to the tumor cell surface, which stimulates tumor phagocytosis. The therapeutic effect is further amplified by simultaneous application of agonists of Toll-like receptors. We searched for ligands that induce both a strong therapeutic effect and are safe for humans.

**Methods:**

B16-F10 murine melanoma model was used. For the stimulation of phagocytosis, mannan or N-formyl-methionyl-leucyl-phenylalanine, was covalently bound to tumor cells or attached using hydrophobic anchor. The following agonists of Toll-like receptors were studied: monophosphoryl lipid A (MPLA), imiquimod (R-837), resiquimod (R-848), poly(I:C), and heat killed *Listeria monocytogenes*.

**Results:**

R-848 proved to be the most suitable Toll-like receptor agonist for our novel immunotherapeutic approach. In combination with covalently bound mannan, R-848 significantly reduced tumor growth. Adding poly(I:C) and *L. monocytogenes* resulted in complete recovery in 83% of mice and in their protection from the re-transplantation of melanoma cells.

**Conclusion:**

An efficient cancer treatment results from the combination of Toll-like receptor agonists and phagocytosis stimulating ligands bound to the tumor cells.

**Electronic supplementary material:**

The online version of this article (doi:10.1186/s12885-016-2982-x) contains supplementary material, which is available to authorized users.

## Background

Cancer immunotherapy based on the stimulation of innate immunity has a long history. W. Coley initiated the first studies at the end of 19^th^ century, using a mixture of inactivated bacteria, Gram-positive *Streptococcus pyogenes* with Gram-negative *Serratia marcescens* - so called Coley’s toxin [1]. Further improvement of cancer immunotherapy based on the use of microorganisms and their parts was significantly influenced by the discovery of pathogen associated molecular patterns (PAMPs). PAMPs allowed for the understanding of mechanisms, how innate immunity recognizes foreign microorganisms, and how the immune response is triggered. Synthetic PAMPs analogues (mainly agonists of Toll-like receptors) were synthesized and tested in cancer therapy [2]. However, the impact of these therapies was not as strong as expected [3]. Even though agonists of Toll-like receptors (TLR) stimulate inflammation, we hypothesize that the infiltrating cells cannot recognize tumor cells as a target of their attack, because they do not have any PAMPs on their surface.

This problem was solved in our previous studies [4, 5], where we described the use of phagocytic receptors agonists anchored to the surface of tumor cells for cancer immunotherapy. To achieve a sufficiently strong therapeutic effect, it was necessary to combine this therapy with simultaneous application of lipopolysaccharide (LPS) – the agonist of TLR4. The obtained synergy resulted in shrinkage and temporary or permanent disappearance of the tumors.

LPS is well tolerated by rodents, dogs and cats [6], but causes septic shock in humans [7]. Therefore we searched for an effective TLR agonist that would be safe for humans and shows strong synergy with phagocytosis stimulating ligands attached to tumor cells. Anchored mannan was selected for the stimulation of phagocytosis, as it gave the best results (mainly survival prolongation) in previous experiments [4, 5]. Resiquimod (R-848) proved to be the best TLR agonist demonstrating synergy with mannan anchored to tumor cells. This innate immunity based cancer immunotherapy was further improved by our search to find the optimal therapeutic mixture, concentration, and timing.

## Methods

### Chemicals

Tissue culture media and media supplements, mannan (S*accharomyces cerevisiae),* laminarin (*Laminaria digitata)*, Tris(2-carboxyethyl)phosphine hydrochloride (TCEP), f-MLF (N-Formyl-methionyl-leucyl-phenylalanine), epicatechin, polyinosinic:polycytidylic acid, sodium salt (poly (I:C)), lipopolysaccharide (LPS) from *Escherichia coli*, TNF-alpha, and GM-CSF were obtained from Sigma-Aldrich (St. Louis, USA). 4-(*N*-Maleimidomethyl) cyclo-hexanecarboxylic-acid N-hydroxysuccinimide ester (SMCC) was provided by Thermo Scientific (Erembodegem, Belgium). Biocompatible Anchor for cell Membrane (BAM, Mw 4000) was obtained from NOF EUROPE (Grobbendonk, Belgium). N-Formyl-methionyl-leucyl-phenylalanine with two lysine molecules (f-MLFKK) was synthesized by Schafer-N (Copenhagen, Denmark). Imiquimod (R-837) was delivered by Merck Millipore (Billerica, USA), monophosphoryl lipid A (MPLA) by Avanti Polar Lipids (Alabaster, USA), and resiquimod (R-848) by Tocris Bioscience (Bristol, UK).

### Cell lines, bacteria and mice

Murine melanoma B16-F10 cells were purchased from American Type Culture Collection (ATCC, Manassas, VA) and were cultivated in RPMI 1640 (Sigma-Aldrich, USA) supplemented with 10% fetal calf serum (FCS, PAA, Austria) and antibiotics. Cells were maintained at 37 °C in a humidified atmosphere with 5% carbon dioxide.

Heat killed *Listeria monocytogenes* was purchased from InvivoGen (Toulouse, France). SPF C57BL/6 mice (female, 18–20 g) were obtained from Charles River Laboratories (Sulzfeld, Germany). Mice were housed in barrier facilities with free access to sterile food and water. Photoperiod was 12/12, temperature 22 °C. All experimental mouse procedures were performed in accordance with the laws of the Czech Republic. Experimental project was approved by the Ministry of Education, Youth and Sports (protocol no. 28842/2014-3).

### Synthesis of mannan-BAM and f-MLFKK-BAM

First, aminated mannan was prepared by reductive amination according to Torosantucci et al. [8]. A mannan solution in an environment of ammonium acetate was reduced by natrium cyanoborohydride at pH of 7.5 and 50 °C for 5 days. The solution was subsequently dialyzed (MWCO 3500 dialysis tubing, Serva, Heidelberg, Germany) against PBS at 4 °C overnight. Peptide f-MLFKK already contained an amino group required for binding of BAM.

Binding of BAM anchor on amino group of mannan (f-MLFKK) was performed at pH of 7.3 [9]. During one hour at room temperature, N-hydroxysuccinimide (NHS) group of BAM reacted with amino group of mannan or with ε-amino group of lysine, respectively.

### Synthesis of mannan-SMCC and * Listeria monocytogenes*-SMCC. In vivo application

Binding of NHS group of heterobifunctional compound SMCC to amino groups of aminated mannan and *Listeria monocytogenes* was performed according to SMCC manufacturer’s instructions (Thermo Scientific, Pierce Protein Biology Products). Binding of mannan-SMCC or *Listeria monocytogenes*-SMCC to tumor cells requires the presence of –SH groups on these cells. Addition of –SH groups on tumor cells was accomplished by the reduction of cystines as previously described [10]. A reducing agent (50 mM solution of TCEP in PBS) was injected intratumorally (i.t.) 1 h prior to application of SMCC ligands. The injection of TCEP alone does not have any effect on tumor growth [4].

### Tumor transplantation

B16-F10 melanoma cells, suspended in serum free RPMI 1640, were inoculated subcutaneously (s.c.) in the previously shaved right flank of mice. Each mouse received 4 × 10^5^ melanoma cells in 0.1 ml of medium.

### Treatment and evaluation of treatment

Randomization of mice in groups was performed 12 days after transplantation of melanoma B16-F10 cells and was immediately followed by initiation of therapies based on intratumoral application of 50 microliters of corresponding preparations (day 0). All mice were housed individually during therapy.

Tumor size was measured with callipers every other day. Tumor volume was calculated, as previously described [11], using the formula V = π/6 AB^2^; A = the largest dimension of tumor mass (length), B = the smallest dimension of the tumor mass (height).

### Mean reduction of tumor growth (%)

The calculation of mean reduction of tumor growth was performed as previously described [4]. After therapy began, on days 4, 6, 8, 10, 12 and 14, the reduction of tumor growth was calculated using the following formula:

$$ \frac{\left(\mathrm{mean}\ \mathrm{tumor}\ \mathrm{volume}\ \mathrm{in}\ \mathrm{control}\ \mathrm{group}\ \hbox{--}\ \mathrm{mean}\ \mathrm{tumor}\ \mathrm{volume}\ \mathrm{in}\ \mathrm{treated}\ \mathrm{group}\right)\ \mathrm{x}\ 100}{\mathrm{mean}\ \mathrm{tumor}\ \mathrm{volume}\ \mathrm{in}\ \mathrm{control}\ \mathrm{group}} $$

The average of calculated reductions in the indicated days is regarded as “mean reduction of tumor growth”.

### Analysis of cell infiltrate using flow cytometry. Cytokine assay

Analysis of cell infiltrate was performed as previously described [4]. Mice were euthanized via cervical dislocation, and the tumors were excised. Subsequently, each tumor was gently washed with cold RPMI 1640 medium, cut into small pieces, and placed into 1 ml cold RPMI 1640 containing 0.33 mg/ml Liberase DL and 0.2 mg/ml DNase I (both Roche Diagnostics, Germany). After a 1 h incubation on a rotary shaker at 37 °C, clumps of tissue aggregates were centrifuged at 160 *g* for 10 min at 4 °C. Supernatant was used to determine IFN-gamma and IL-10 using the ELISA kit (eBioscience and LSBio, respectively), performed according to manufacturers recommendations. The resulting pellet was gently passed through a plastic strainer (70 μm, BD Biosciences, USA) into cold PBS (pH 7.3) and washed by centrifugation at 160 *g* for 10 min at 4 °C. Cells were then transferred into a 96-well plate (Corning Incorporated, USA) and analyzed by flow cytometry. Particular leukocyte subtypes were determined using the following monoclonal antibodies (eBioscience, USA): a) Total leukocytes - anti-mouse CD45 PerCP-Cy5.5; clone 30-F11, b) T cells - anti-mouse CD3e FITC; clone 145-2C11, c) CD4+ T cells - anti-mouse CD4 APC; clone GK1.5, d) CD8+ T cells - anti-mouse CD8a; clone 53–6.7, e) B cells - anti-mouse CD19 APC; clone eBio1D3, f) NK cells - anti-mouse NK1.1 PE; clone PK136, g) granulocytes (anti-mouse Ly-6G (Gr-1) Alexa Fluor 700; clone RB6-8C5, h) macrophages - anti-mouse F4/80 Antigen PE-Cy7; clone BM8, and i) dendritic cells - anti-Mouse CD11c PE; clone N418, anti-Mouse MHCII (I-A/I-E) Alexa Fluor 700; clone M5/114.15.2). Analysis was performed using a BD FACSCanto II flow cytometer (BD Biosciences, USA), equipped with two lasers (excitation capabilities at 488 nm and 633 nm). BD FACSDiva software 6.1.3. was used for the analysis of flow cytometry data.

### Preparation and priming of neutrophils

Neutrophils were isolated from murine bone marrow according to Stassen et al. [12] and subsequently purified using MACS technique (Miltenyi Biotec). Purity was checked by BD FACSCanto II flow cytometer (BD Biosciences, USA) using anti-mouse CD45 APC, Clone: 30-F11 and anti-mouse Ly-6G (Gr-1) Alexa Fluor 700, Clone: RB6-8C5 antibodies (eBioscience). Neutrophils were primed according to Dewas et al. [13] by the mixture of GM-CSF and TNF-alpha (12 ng and 2.5 ng/ml respectively) for 20 min. The priming solution was enriched with 2 micromolar solution of soluble beta glucan (laminarin) as previously described [5]. Experiments were performed in complement containing medium (FCS was not heat inactivated).

### Statistical analysis

Statistical analysis was performed using one-way ANOVA with Tukey’s *post hoc* test and Log-rank test, respectively (STATISTICA 12, StatSoft, Inc., Tulsa, OK 74104, USA). Error bars indicate SEM.

## Results

### Searching for proper combination of TLR agonist and phagocytosis stimulating ligand leading to effective melanoma B16-F10 immunotherapy

The main goal of our study was to find proper TLR agonist(s), which, in combination with phagocytosis stimulating ligands, would result in tumor shrinkage and elimination. Previously, we discovered that mannan attached to tumor cells (hydrophobic BAM anchor or SMCC) was a good stimulator of phagocytosis [4, 5]. Thus, we used this finding throughout the present study. TLR ligand replacement was necessary due to the previously used LPS, a TLR4 agonist, which poses a dangerous threat to humans [4, 5]. Overall, three different TLR agonists were tested as possible LPS replacements.

**Fig. 1:**
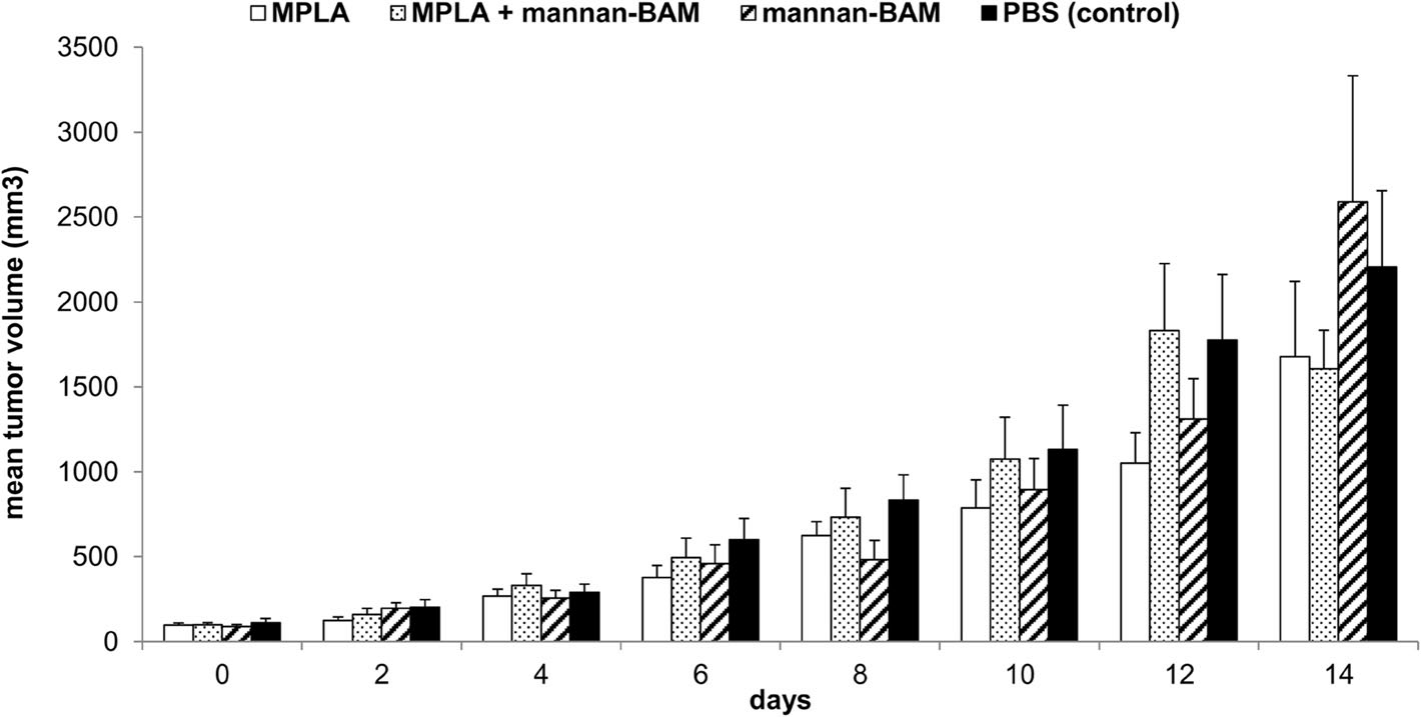
Immunotherapy of melanoma B16-F10 using MPLA alone or in combination with mannan-BAM. C57BL/6 mice (females) were inoculated subcutaneously in a shaved area of the right flank with 4 × 10^5^ murine melanoma B16-F10 cells per mouse in 0.1 ml RPMI. Twelve days after tumor transplantation, mice were randomized in groups of six. Therapies started immediately. The preparations were applied intratumorally (50 microliters/mouse) in pulse regime (days 0, 1, 2…8, 9, 10). After therapy commenced, mice were kept individually. Tumors were measured every second day for 14 days and their volume was calculated. The composition of preparations used was: 0.5 mg MPLA/ml PBS, 0.5 mg MPLA/ml 0.2 mM mannan-BAM in PBS, 0.2 mM mannan-BAM in PBS, PBS

First, we tried monophosphoryl lipid A (MPLA) which is an LPS derivative and TLR4 agonist with very low toxicity for humans. However, MPLA, mannan-BAM, or their mixture, lead to only slight, non-significant tumor growth reduction (Fig. 1). No signs of MPLA and mannan-BAM synergy were observed. Similar results were observed with the use of another tested compound, TLR7 agonist imiquimod (R-837) (data not shown).

**Fig. 2:**
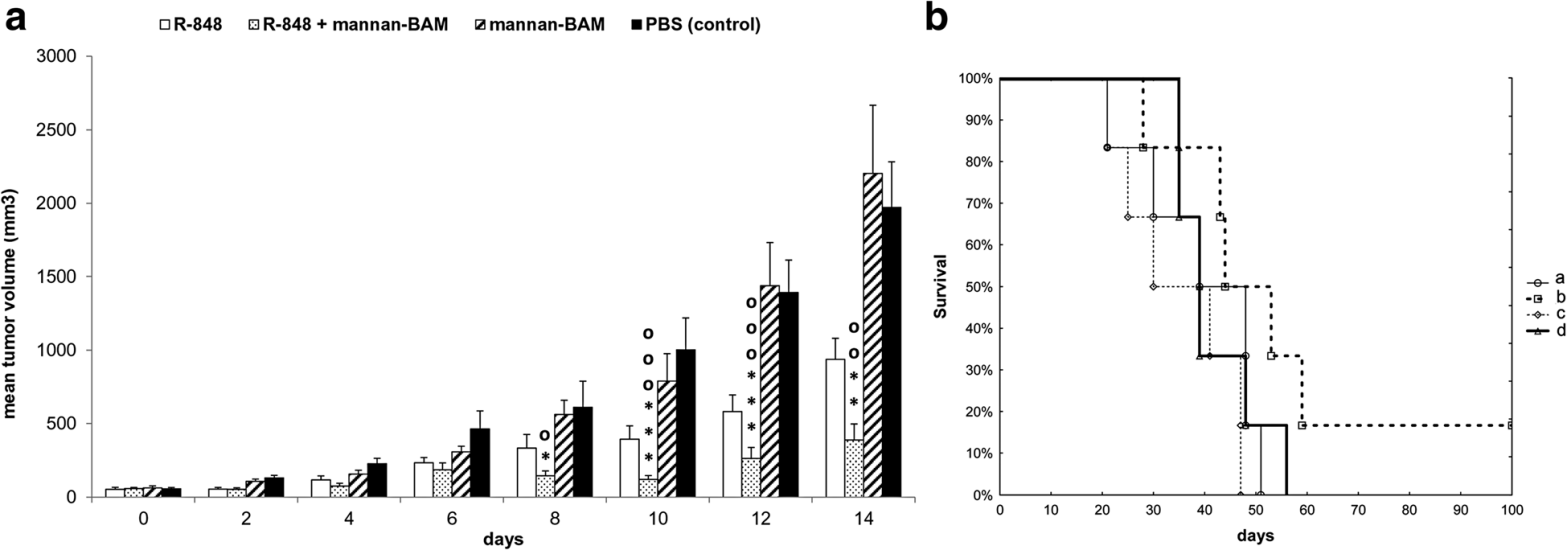
Immunotherapy of melanoma B16-F10 based on the synergy of R-848 and mannan-BAM. The experimental design was the same as described in Fig. 1. Six mice were used per group. The composition of therapeutic mixture was: 0.5 mg R-848, HCl form/ml PBS, 0.5 mg R-848, HCl form/ml 0.2 mM mannan-BAM in PBS, 0.2 mM mannan-BAM in PBS, PBS. **a** The effect of therapy on tumor growth. * *P* ≤ 0.05 * * *P* ≤ 0.005 * * * *P* ≤ 0.0005 compared to control (PBS). o *P* ≤ 0.05 o o *P* ≤ 0.005 o o o *P* ≤ 0.001 compared to mannan-BAM. **b** Survival analysis. a – R-848, b – R-848 + mannan-BAM, c – mannan-BAM, d- PBS (control)

Resiquimod (R-848), a TLR7 agonist in mice and TLR7 and 8 agonists in humans, was likewise studied. The R-848 + mannan-BAM combination revealed a strong synergistic effect resulting in 75.4% mean reduction of tumor growth (Fig. 2a). As shown in Fig. 2b, mice treated with this combination survived longer than PBS treated control group. However, the difference was not statistically significant. This experiment was repeated twice with similar results, including the observation of more than 100 days survival.

### Therapy based on combination of R-848 with anchored f-MLF motif

**Fig. 3:**
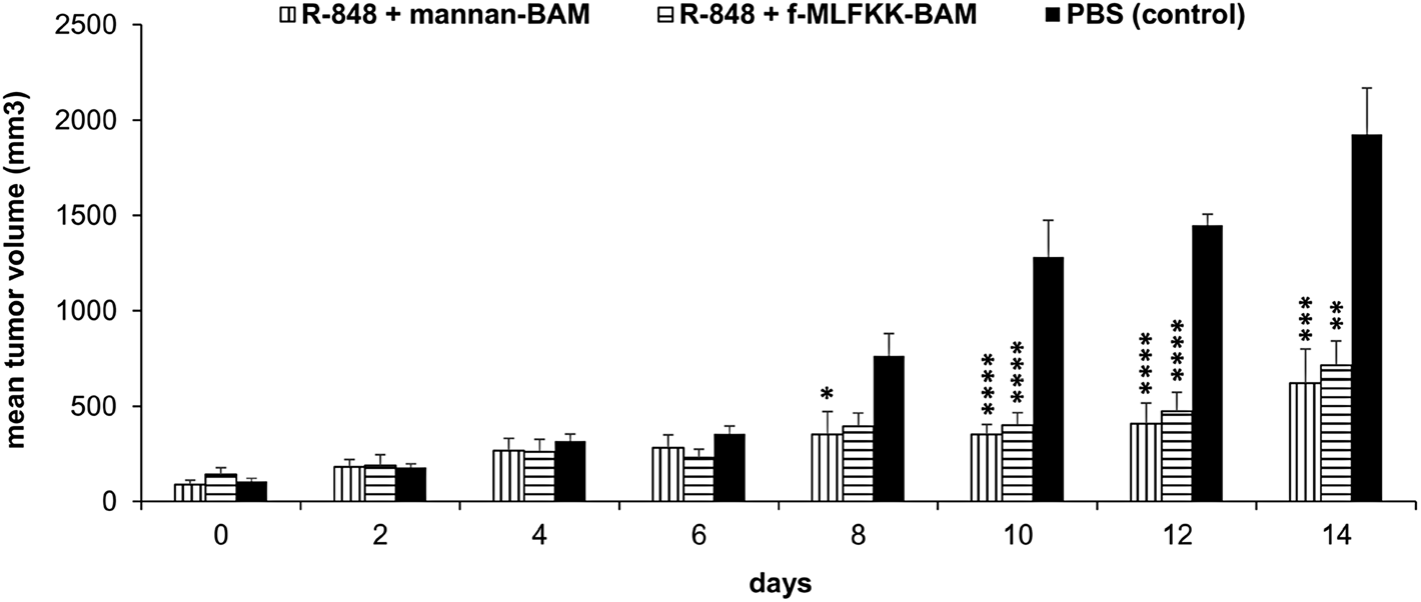
Therapy based on the combination of R-848 with anchored f-MLF motif. The experimental design was the same as described in the Fig. 1. Six mice were used per group. The composition of therapeutic mixture was: 0.5 mg R-848, HCl form/ml 0.2 mM mannan-BAM in PBS, 0.5 mg R-848, HCl form/ml 0.5 mM f-MLFKK-BAM in PBS, PBS was used as a control. * *P* ≤ 0.05 ** *P* ≤ 0.005 *** *P* ≤ 0.001 **** *P* ≤ 0.0005 compared to control (PBS)

The effect of therapeutics based on anchored mannan depends on the presence of mannan binding lectin (MBL) in serum. As 5–10% of humans lack MBL, it is necessary to have mannan independent therapeutic system. Therefore, we tested the combination R-848 + f-MLFKK-BAM and compared it to R-848 + mannan-BAM. Both mixtures caused comparable reduction of tumor growth (Fig. 3).

### Immunotherapy of melanoma B16-F10 based on the synergy of R-848 and mannan-SMCC. Further improvement using poly(I:C) and anchored *L. monocytogenes*

**Fig. 4:**
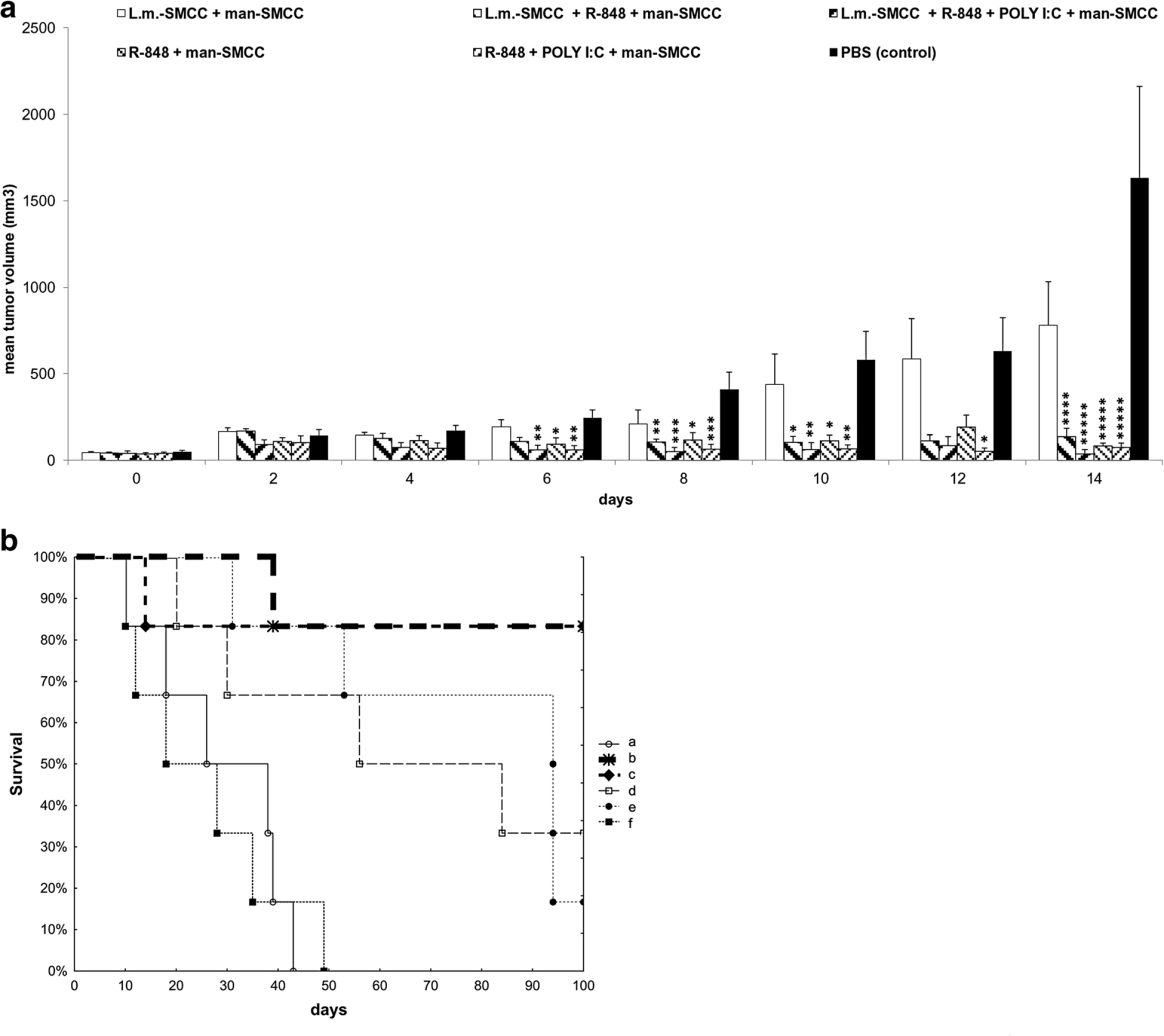
Immunotherapy of melanoma B16-F10 based on the synergy of R-848 and mannan-SMCC. Further improvement using poly(I:C) and anchored *L. monocytogenes*. Tumor transplantation and measurement was performed as described in Fig. 1. Six mice were used per group. Therapies, based on intratumoral application of corresponding preparations (50 microliters/mouse), started 12 days after tumor transplantation. Four therapeutic pulses were applied on days 0, 1, 2…8, 9, 10…16, 17, 18…24, 25, 26. Therapeutic mixture contained following concentrations of active parts dissolved in PBS: 1 billion *L. monocytogenes*-SMCC/ml, 0.5 mg R-848, HCl form/ml, 0.5 mg poly(I:C)/ml, 0.2 mM mannan-SMCC, PBS was used as control. **a** The effect of therapy on tumor growth. * *P* ≤ 0.05 ** *P* ≤ 0.01 *** *P* ≤ 0.005 **** *P* ≤ 0.001 ***** *P* ≤ 0.0005 compared to control (PBS). **b** Survival analysis. a – L.m.-SMCC + man-SMCC. b – L.m.-SMCC + R-848 + man-SMCC. c – L.m.-SMCC + R-848 + poly(I:C) + man-SMCC. d – R-848 + man-SMCC. e – R-848 + poly(I:C) + man-SMCC. f – PBS (control). b versus f ….. *P* ≤ 0.005. c versus f ….. *P* ≤ 0.01. d versus f ….. *P* ≤ 0.05. e versus f ….. *P* ≤ 0.05

The combination of R-848 with anchored mannan showed the best therapeutic effect from all studied combinations. Thus, we focused on further improvement of this therapy. Specifically, we tested a stronger binding of mannan-SMCC (covalent binding) together with the addition of other TLR agonists. *Listeria monocytogenes*, a predominant agonist of TLR2, was added alone and/or in the combination with TLR3 agonist poly(I:C). As shown in Fig. 4a, the mixture of R-848 with mannan-SMCC, resulted in strong inhibition of tumor growth. Further addition of *L. monocytogenes* alone or in the combination with poly(I:C) did not significantly improve its therapeutic effect. The major effects of additives were observed in the survival experiments. As shown in Fig. 4b, groups treated with *L. monocytogenes* exhibited significantly higher survival rates than untreated groups. An 83.3% survival rate was observed, independent of the presence or absence of poly(I:C). Furthermore, all surviving mice were re-transplanted again with B16-F10 on day 120. As shown in Table 1, re-transplantation was successful in one mouse in the group b and in two mice in group d. This resulted in death of animals. In the groups treated with the mixture containing poly(I:C) (c and e), the mice were fully protected against re-transplantation. All mice lived without any pathological symptoms for more than 1 year after treatment.

**Table 1:** Immunotherapy of melanoma B16-F10 based on the synergy of R-848 and mannan-SMCC. Further improvement using poly(I:C) and anchored *L. monocytogenes*. Re-transplantation

Initial treatment	Number of survived mice	Group (see Fig. 4b)	Number of successful re-transplantations
*L. monocytogenes*-SMCC + R-848 + mannan-SMCC	5	b	1
*L. monocytogenes*-SMCC + R-848 + poly(I:C) + mannan-SMCC	5	c	0
R-848 + mannan-SMCC	2	d	2
R-848 + poly(I:C) + mannan-SMCC	1	e	0

Re-transplantation of mice that survived in experiment shown in Fig. 4 was performed on day 120. All surviving mice were inoculated again with B16-F10 (4 × 10^5^ melanoma cells/mouse s.c.)

### Flow cytometry analysis of cell infiltrate in R-848 + poly(I:C) + *L. monocytogenes*-SMCC + mannan-SMCC melanoma treatment. Cytokine assay

**Fig. 5:**
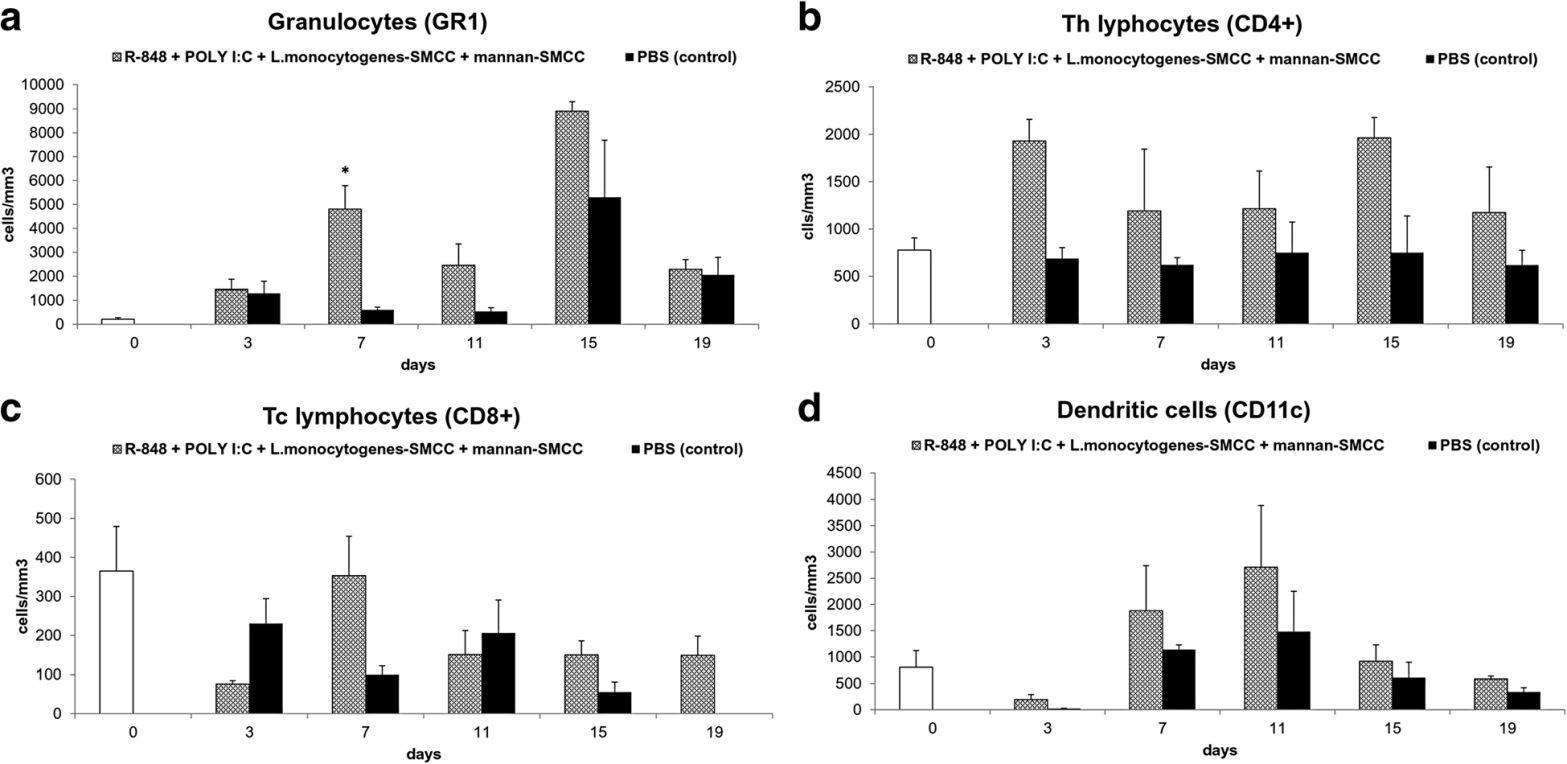
Flow cytometry analysis of cell infiltrate in R-848 + poly(I:C) + *L. monocytogenes*-SMCC + mannan-SMCC melanoma treatment. The transplantation of melanoma B16-F10 was performed as described in Fig. 1. Twelve days after tumor transplantation, mice were randomized in two groups of 24. Therapy based on intratumoral application of corresponding therapeutic mixtures (50 microliters/mouse) started immediately. Four therapeutic pulses were applied on days 0, 1, 2…8, 9, 10…16, 17, 18…24, 25, 26. The composition of therapeutic mixture was: 1 billion *L. monocytogenes*-SMCC + 0.5 mg R-848, HCl form + 0.5 mg poly(I:C)/ml 0.2 mM mannan-SMCC in PBS, PBS was used as a control. Three mice from each group were euthanized on days 3, 7, 11, 15, 19 after the start of the therapy. Three mice were killed without any application at time 0 (negative control). The analysis of cell infiltrate of excised tumors was performed using flow cytometry and expressed as cells/mm^3^ of tumor mass. The following labeled antibodies were used: **a** anti-mouse Ly-6G (Gr-1) Alexa Fluor 700 (granulocyte detection), **b** anti-mouse CD4 APC; clone GK1.5 (CD4+ Th lymphocytes), **c** anti-mouse CD8a; clone 53–6.7 (CD8+ Tc lymphocytes), **d** anti-Mouse CD11c PE; clone N418, anti-Mouse MHCII (I-A/I-E) Alexa Fluor 700; clone M5/114.15.2 (dendritic cells). * *P* ≤ 0.05 compared to control (PBS)

During the course of therapy with the complex therapeutic mixture (R-848 + poly(I:C) + *L. monocytogenes*-SMCC + mannan-SMCC), which showed the best therapeutic effect in the previous experiment, we analyzed tumor infiltrate from treated mice and compared it with a PBS control. In treated group, a strong granulocytic infiltration was observed. In particular, infiltration was higher between days 7 and 15, reaching statistical significance on day 7 (Fig. 5a). Minor, but not significant, increase of CD4+ Th lymphocytes was observed in the treated group, which contrasts with the no change observed in the control group (Fig. 5b). The levels of Tc lymphocytes (CD8+) were low in both groups throughout the monitored period (Fig. 5c). No dramatic changes in the count of dendritic cells were observed. However, a non-significant, higher quantity of these cells was observed throughout the monitored period when compared to the control group (Fig. 5d). No changes in B lymphocyte, NK, and monocyte/macrophage counts were observed.

**Fig. 6:**
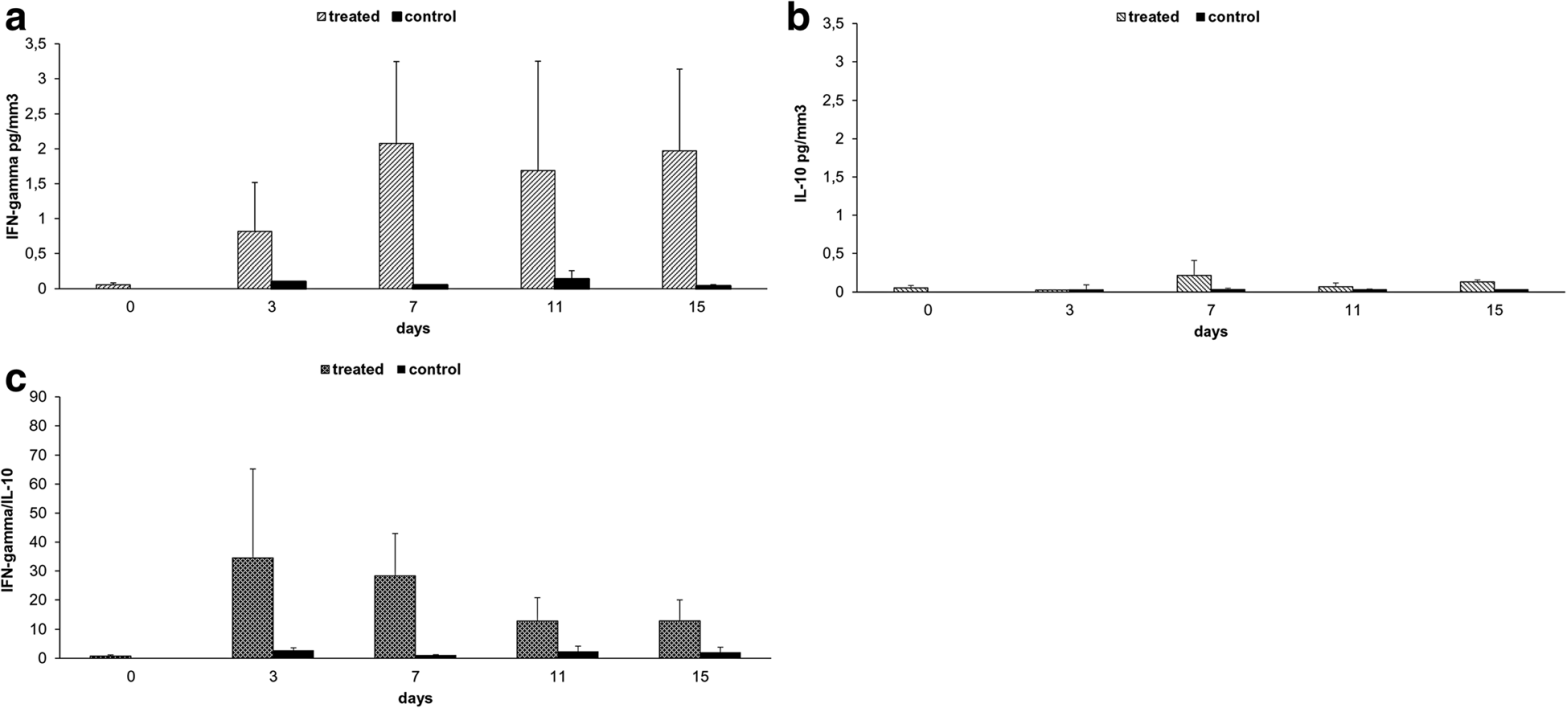
R-848 + poly(I:C) + *L. monocytogenes*-SMCC + mannan-SMCC melanoma treatment. Cytokine assay. After separation of cells from tumors excised in previous experiment (Fig. 5), cytokine analysis of corresponding supernatants was performed: **a** IFN-gamma, **b** IL-10, **c** calculated ratio of IFN-gamma/IL-10

Cytokines measurement revealed high levels of IFN-gamma (Fig. 6a), low levels of IL-10 (Fig. 6b), and high IFN-gamma/IL-10 ratio (Fig. 6c) in tumor environment of treated mice indicating initiation of Th1 response.

At the beginning of the therapy, mean tumor volume was 155.4 + −93.2 mm^3^. Analysis of tumor infiltrating cells and cytokines was terminated on day 19 of treatment, as 9 surviving mice were tumor free.

### Interaction of neutrophils with opsonized tumor cells – frustrated phagocytosis and oxidative burst

The role of phagocytes (granulocytes) in the herein described cancer treatment approach based on artificial opsonization of tumor cells is supported in Fig. 5. Depletion of neutrophils by Ly6G antibody reduced the effect of R-848 + mannan-BAM therapy [unpublished results]. An attempt of phagocytes (especially neutrophils) to phagocyte relatively large melanoma cells was described as a specific type of frustrated phagocytosis [5, 14]. This idea was supported by the estimation of the frequency of frustrated phagocytosis events in neutrophil-melanoma interaction, observed during in vitro experiments (Table 2). The key role of mannan and f-MLF attachment to tumor cell surface for the stimulation of frustrated phagocytosis was demonstrated.

**Table 2:** Interactions between neutrophils and melanoma cells with or without anchored ligands of phagocytic receptors

	Mean count of neutrophils attached to one melanoma cell by mechanism of frustrated phagocytosis		
Ligand	20 min.	30 min.	40 min.
Mannan-BAM	0.333	0.556	0.733
Mannan free	0.111	0.000	0.000
f-MLFKK-BAM	0.589	0.311	0.360
f-MLF free	0.480	0.111	0.000
PBS (control)	0.000	0.000	0.000

B16-F10 melanoma cells were incubated (30 min, 37 °C) with 0.02 mM mannan-BAM or 0.05 mM f-MLFKK-BAM in culture medium and then subsequently washed. Suspension of bone marrow neutrophils (90% purity) primed with GM-CSF + TNF-alpha (+ laminarin in case of mannan-BAM) in culture medium was added to B16-F10 cells. Free ligands were added in a concentration of 0.02 mM (mannan) and 0.05 mM (f-MLFKK). Neutrophils and melanoma cells were incubated at a 2:1 ratio

The rate of frustrated phagocytosis events was estimated by light microscopy: Frustrated phagocytosis was defined as neutrophil/melanoma cell contact, where neutrophil adhere tightly to B16-F10 cell. Such contact is further characterized by neutrophil flattening and gaining of waning moon shape [5]

**Fig. 7:**
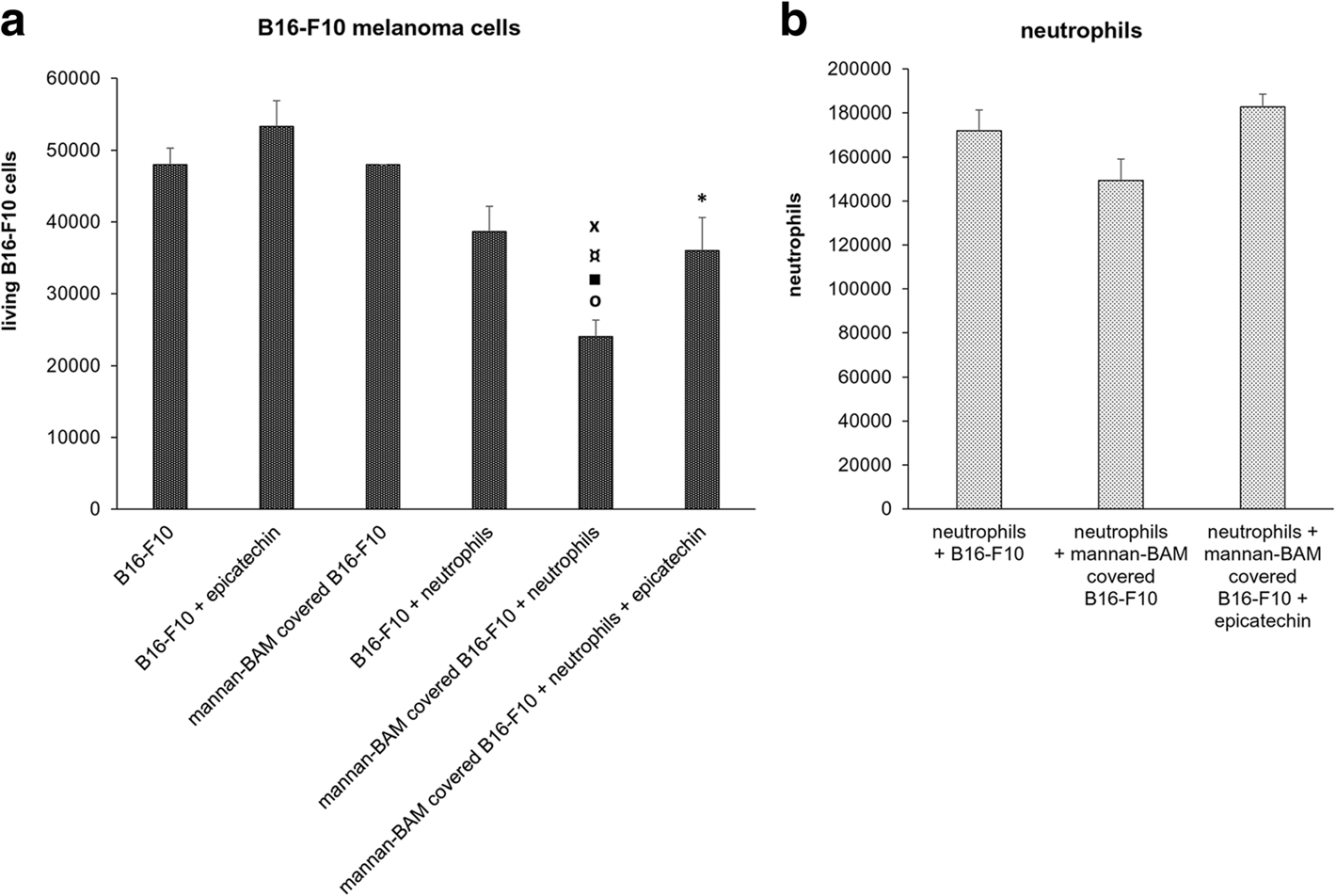
The interaction of neutrophils with opsonized melanoma cells. Oxidative burst. B16-F10 melanoma cells were incubated (30 min, 37 °C) with 0.02 mM mannan-BAM in culture medium and subsequently washed. The suspension of bone marrow neutrophils (90% purity), primed with GM-CSF + TNF-alpha + laminarin in culture medium was added to B16-F10 (both free and mannan-BAM covered) in the ratio 5:1. Where indicated, 0.1 mM epicatechin was added. All mixtures were incubated for 2 hours at 37 °C. After the incubation, living, trypan blue excluding melanoma cells (**a**) and neutrophils (**b**) were counted with a haemocytometer. *P ≤ 0.05 compared to B16-F10 + epicatechin. o*P* ≤ 0.005 compared to B1-F10. ■*P* ≤ 0.0005 compared to B16-F10 + epicatechin. ¤*P* ≤ 0.005 compared to mannan-BAM covered B16-F10. x*P* ≤ 0.05 compared to B16-F10 + neutrophils

Frustrated phagocytosis is initiated by tight contact between neutrophils and melanoma cells and is followed by the release of granule content into the pockets formed between neutrophils and tumor cells [5, 14]. Granules contain components involved in killing target melanoma cells either directly (hydrolases, defensins) or indirectly (myeloperoxidase dependent HClO formation connected with oxidative burst). We analyzed the cytotoxic effect of these processes and the participation of oxidative burst dependent mechanisms. The latter was analyzed using epicatechin - an inhibitor of oxidative burst. As shown in Fig. 7a, neutrophils killed 50% of opsonized melanoma cells and the inhibition of oxidative burst resulted in 50% reduction of cytotoxicity. This result supports the hypothesis that oxidative burst participates on cytotoxic effects resulting from the frustrated phagocytosis.

Neither melanoma cells (Fig. 7a) nor neutrophils (Fig. 7b) were directly affected by epicatechin alone.

## Discussion

Our previous study demonstrated that TLR agonists combined with phagocytic receptors ligands may act as an effective cancer therapy [4, 5]. In the present follow-up study, we focused on searching for ligands that could be applied in humans. To stimulate phagocytosis, mannan was attached to tumor cells either covalently by an SMCC anchor or through a hydrophobic BAM anchor. As we described previously, mannan stimulates MBL-dependent phagocytosis, which kills tumor cells [4, 5]. This is based on the initiation of the lectin pathway of complement activation with MBL-mannan complex, leading to iC3b opsonization of target cells.

The TLR4 agonist LPS, which was used in our previous studies [4, 5] cannot be utilized in humans due to the risk of developing septic shock. We tested several possible replacement compounds, the first being MPLA, a low-toxicity derivate of the lipid A region of LPS [15]. MPLA alone showed only negligible effect on tumor growth. When combined with mannan, no synergy resulted. Mata-Haro and colleagues [16] reported that low toxicity of MPLA, in comparison to LPS, is caused by the active suppression of pro-inflammatory activity. This could explain the failure of MPLA + mannan-BAM therapy, as infiltration of inflammatory cells is crucial for the presented therapy [4, 5].

R-837, another TLR agonist, has been shown to induce an anti-tumor immune response and is being used to treat skin tumors [17]. However, in our experiments, R-837 only exhibited a weak effect, and was thus not involved in further treatments. An explanation for the insufficient impact of R-837 on tumor cells could be that it induces less pronounced production of cytokines and enhancement of cellular immunity than R-848, for a review see [18].

The last tested LPS replacement substance was R-848. R-848 alone caused visible, but not statistically significant tumor growth reduction. Complete recovery was not observed. However, R-848 combined with anchored mannan resulted in significant synergy and partial recovery of treated mice. Regarding the mechanisms of action, we are considering the important role of granulocytes (neutrophils), as their strong infiltration was noticed. In our previous in vitro experiments, we observed a significant cytotoxic effect of neutrophils against the tumor cells opsonized with mannan. These tumor cells were killed by frustrated phagocytosis [5]. In herein present study, we confirmed our previous observations and by using epicatechin, revealed significant participation of oxidative burst in killing mechanisms.

The synergy between R-848 and anchored mannan corresponds to our therapeutic concept based on inflammatory infiltration of tumors and the direction of recruited phagocytes to opsonized tumor cells [4, 5]. Ensuring proper timing of drug delivery is vital for an effective therapy. For R-848 + mannan based therapies we used the same pulse regime as previously described [4]. The optimal therapeutic scheme corresponds well with the observation from Bourquin et al. [19], who based their tumor treatment strategy on the repeating cycles of R-848 injections, separated by treatment-free intervals. Treatment free intervals are necessary for the recovery of sensitivity to R-848. R-848, like other TLR agonists, induces TLR tolerance, which should be circumvented by proper timing of therapy [20, 21].

The induction of synthesis of pro-inflammatory cytokines in the tumor environment is important for the recruitment of inflammatory cells. Simultaneously, conditions for the shift of tumor-associated macrophages (TAMs) towards an anti-tumor, pro-inflammatory M1 phenotype and reduction of the activity of tumor protecting immunosuppressive T regulatory lymphocytes (Tregs) and myeloid derived suppressor cells (MDSC) are created [22]. The direct effect of R-848 on MDSC count reduction [23] and the stimulation of phagocytic activity of infiltrating cells by TLR agonists should be taken into account [24].

R-848 also induces the maturation of plasmacytoid dendritic cells (pDC) [25] and promotes the production of antibodies [26]. R-848 was described as potential vaccine adjuvant enhancing Th1 response in mice [27]. Furthermore, R-848 also had a direct effect on tumor cells - it upregulates the expression of opioid growth factor receptor, which leads to the anti-proliferative and cancer suppressive effects, independent of immune function [28]. All these mechanisms can contribute to the effect of therapy.

Since a small percentage of humans are MBL deficient, f-MLF was tested as an alternative ligand of phagocytic receptors. When anchored, f-MLF was able to stimulate phagocytosis and kill tumor cells [5]. Positive results of the treatment with R-848 + anchored f-MLF supported the possibility of using this ligand for the treatment of patients with an MBL deficiency.

To enhance the effect of R-848 + mannan based therapy, we tested the the effects of adding of heat killed *L. monocytogenes* into the treatment mixture. Introduction of *L. monocytogenes* did not accelerate the shrinkage of tumors, but had a strong effect on survival rate of mice. Heat killed *L. monocytogenes* is able to induce Th1-dominated immune response [29]. We hypothesize that cell-mediated adaptive immunity joined the innate immune response and eliminated the remaining melanoma cells. This is supported by the observed Th1 response initiation. Moreover, 80% of mice were protected against re-transplantation of melanoma cells, which suggests that acquired immunity response was directed against melanoma specific antigens and that tumor antigen-specific memory cells were involved.

The addition of poly(I:C) into the therapeutic mixture (with and without *L. monocytogenes*) also increased the resistance of treated mice against re-transplantation. Poly(I:C) works in synergy with R-848 at the level of stimulation of pro-inflammatory cytokines synthesis [30, 31] and is frequently used as vaccine adjuvant. Additionally, poly(I:C) stimulates both human [32] and murine [33] dendritic cells maturation, so it can enhance antigen presentation to the cells of adaptive immunity.

Survival of all treated mice for more than 1 year after treatment serves as indirect proof that the presented combined therapy (*L. monocytogenes* + R-848 + poly(I:C) + mannan-SMCC) may eliminate metastases as well, because B16-F10 tumors metastasize very early (before the day 10 after transplantation as described by Wald et al. [34], i.e. prior to the initiation of our therapy). However, this aspect needs further investigation.

In summary, we have demonstrated the strong therapeutic effect when the TLR agonist R-848 is combined with anchoring mannan to the tumor cells. This effect was further enhanced by addition of another TLR agonists (poly(I:C), *L. monocytogenes*) into the therapeutic mixture. Innate immunity cells, particularly neutrophils, seem to play a key role in the presented treatment mechanism. Evaluating the role of adaptive immunity in the above described therapy will be the main goal as we continue our research.

## Conclusions

Therapy based on R-848 + mannan-SMCC with supportive *L. monocytogenes* and poly(I:C) is much too complex to provide a detailed description of all involved mechanisms. Nevertheless, the acting components play important roles and perform in synergy. We assume that this therapy can be used for cancer treatment in humans, as the majority of the components in the therapeutic mixture have already been used or tested in clinical trials. The presented treatment of fast growing, aggressive and low immunogenic B16-F10 melanoma, represent a base for promising future research in the field of human cancer immunotherapy.

## Additional files

Additional file 1: Fig1-4,6,7_DATA. (XLSX 45 kb)
Additional file 2: Fig5_DATA. (XLSX 151 kb)

## Abbreviations

f-MLF: N-Formyl-methionyl-leucyl-phenylalanine
f-MLFKK: N-Formyl-methionyl-leucyl-phenylalanine with two lysine molecules
LPS: Lipopolysaccharide
PAMPs: Pathogen associated molecular patterns
poly(I:C): Polyinosinic:polycytidylic acid, sodium salt
SMCC: 4-(*N*-Maleimidomethyl) cyclo-hexanecarboxylic-acid N-hydroxysuccinimide ester
TCEP: Tris(2-carboxyethyl)phosphine hydrochloride
TLR: Toll-like receptor
